# Conjoined Pathways: Unraveling the Coexistence of Developmental Venous Anomaly With Arteriovenous Malformation

**DOI:** 10.7759/cureus.44350

**Published:** 2023-08-29

**Authors:** Sahil Navlani, Akshata Mestha, Amritendu Mukherjee, Ahmad Saadat Abdelmuhdi, Ayman A Sibaie

**Affiliations:** 1 Medicine and Surgery, Dubai Academic Health Corporation, Dubai, ARE; 2 Radiology, Rashid Hospital, Dubai, ARE

**Keywords:** stroke, neuroimaging, digital subtraction angiography, cerebral vascular malformation, developmental venous anomaly, arteriovenous malformation

## Abstract

Developmental venous anomalies (DVAs) are intracranial vascular malformations typically characterized by their benign nature, often obviating the need for radiological follow-up. These anomalies arise from variations in the standard drainage pattern. While previously deemed congenital, there has been ongoing debate about a developmental component contributing to their etiology. They frequently coexist with other cerebral venous malformations (CVM); however, their association with arteriovenous malformations (AVM) is exceedingly rare. Such mixed malformations pose a therapeutic challenge, necessitating meticulous consideration for appropriate treatment. We present a noteworthy case involving a patient with arteriovenous malformation along with dual developmental venous anomalies, one of which served as the draining vein for the AVM.

## Introduction

Previously referred to as venous angiomas, developmental venous anomalies (DVAs) represent significant deviations from the usual trans-medullary veins that play a crucial role in facilitating the drainage of white and gray matter [1,2]. They consist of medullary veins arranged in a radial fashion that unite together to form an enlarged transcortical or subependymal collector vein [2,3]. They are congenital lesions and when present independently, are usually benign and have a low risk for hemorrhage with an estimated annual bleeding rate of 0.2% [2,4]. It has been found to be asymptomatic in up to 50% of cases [4] and may present only incidentally on neuroimaging scans [2]. Frequently observed among young adults, the average age of onset for DVAs accompanied by arteriovenous malformations (AVM) is 31 years [5]. DVAs are one of the most commonly occurring cerebral venous malformations (CVM) but their association with AVM is not very well documented [6]. Although in the past DVAs were considered to be an uncommon phenomenon, nowadays with the advancement in magnetic resonance imaging (MRI), it has been more commonly detected. They usually appear as caput medusae on MRI and cerebral angiography [7]. 

## Case presentation

A 66-year-old male known case of hypertension and dyslipidemia, presented with left-sided body numbness, slurred speech, dizziness, and facial droop for six hours after getting up in the morning. There was no loss of consciousness, vomiting, seizures, or limb weakness. The patient is a chronic smoker and heavy alcohol drinker. The patient was hypertensive with a blood pressure of 190/105 mmHg and the Glasgow Coma Scale (GCS) was 15/15 in the emergency department. On physical examination, there was right upper motor neuron facial palsy, power in upper and lower limbs were 5/5, sensations were intact bilaterally in upper and lower limbs and pronator drift was absent. The National Institutes of Health Stroke Scale (NIHSS) was two. A working diagnosis of minor stroke was made and the patient was given a loaded dose of dual antiplatelet therapy (DAPT) with aspirin 300 mg and clopidogrel 300 mg.

**Figure 1:**
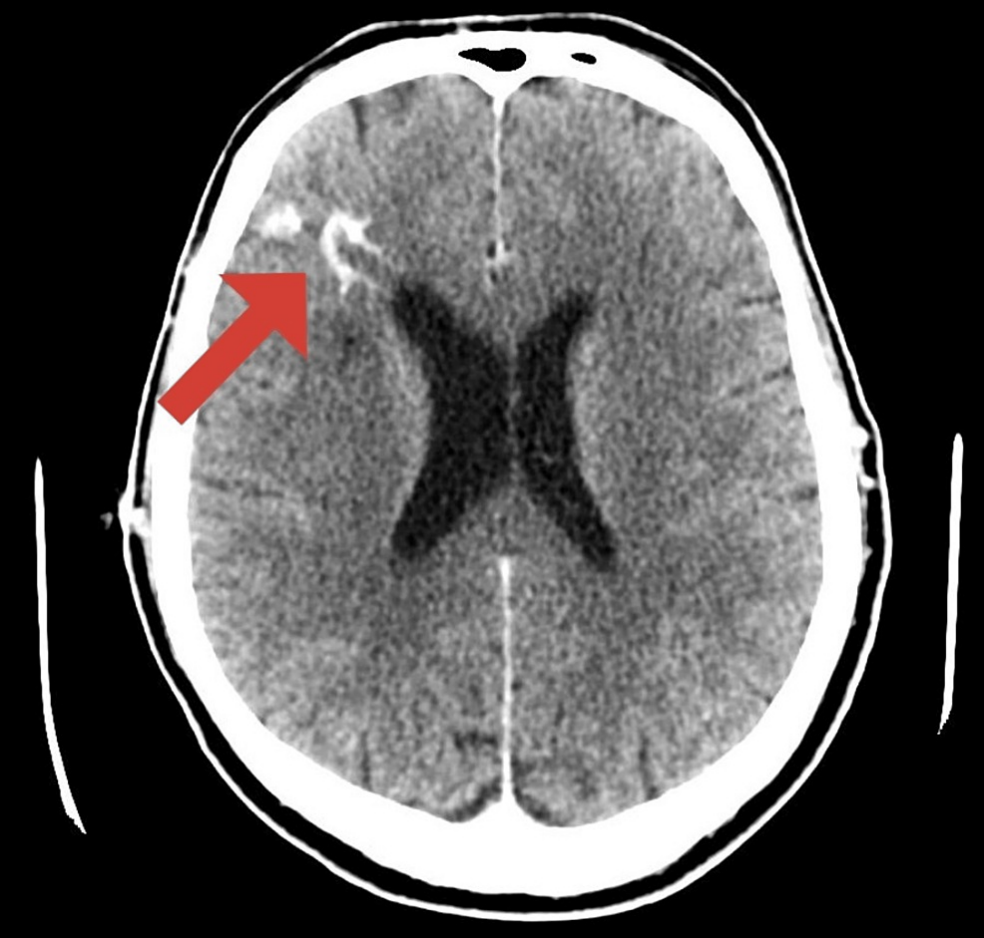
CT Brain with contrast shows a hyperdense area with an abnormal tuft of vessels in the right frontal cortex.

Computed tomography (CT) brain was done and showed old lacunar infarcts at multiple sites. A gyral hyperdensity was noted in the right frontal cortex. There were no intracerebral or extra-cerebral bleeds, acute infarcts, space-occupying lesions, or midline shifts. Multiphase CT brain angiogram (mCTA) was done during peak arterial, peak venous, and late venous phases. Atherosclerotic calcified plaques were noted at multiple sites. There was a hyperdense area with an abnormal tuft of vessels noted in the region of the right frontal cortex with veins draining into an anomalous vein which was draining into the parietal cortical veins and further towards the superior sagittal sinus in the region of the right frontal cortex (Figure 1). Arterial supply could not be delineated due to the presence of a tuft of vessels. There were areas of moderate narrowing at the proximal and distal A3 segment of the anterior cerebral artery on the left side. During the peak venous and late venous phases, there was no delay in opacification of the peripheral cortical cerebral veins. There was good collateral, leptomeningeal vascular backfilling of the arteries, and normal venous drainage bilaterally.

In view of the enhanced tuft of vessels on the arterial phase of mCTA with linear vascular channels seen extending to the right frontal dural surface, the possibility of the dural AV fistula was suspected. Hence, evaluation by a conventional angiogram was to be carried out. In the meanwhile, DAPT was changed to a single antiplatelet with aspirin 75 mg in view of the AVM.

**Figure 2:**
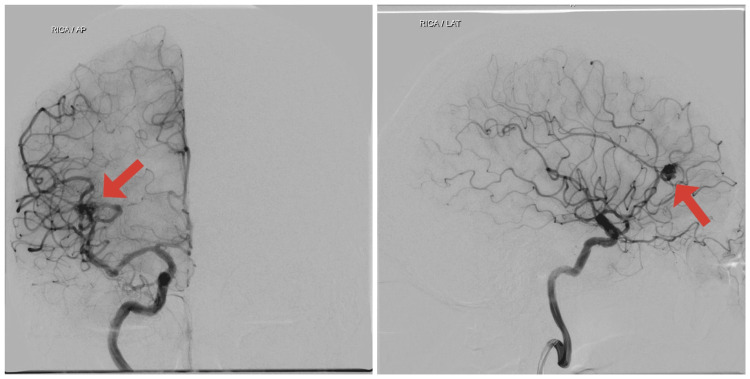
An arteriovenous malformation (AVM) nidus with an arterial feeder from the middle cerebral artery (MCA) is seen on anteroposterior (AP) and lateral view of digital subtraction angiography (DSA) on the right internal carotid artery (ICA) injection.

**Figure 3:**
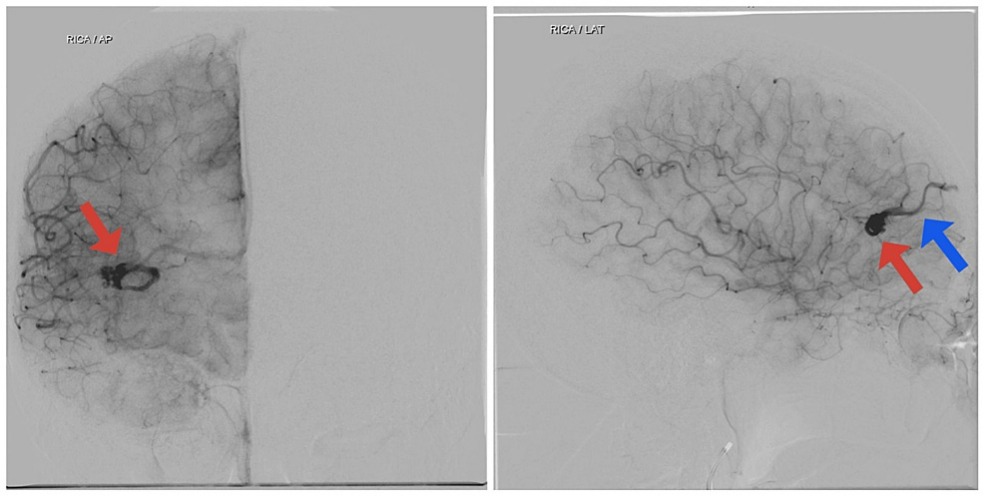
Arteriovenous malformations (AVM) nidus (red arrow) with its draining vein (blue arrow) is seen on anteroposterior (AP) and lateral view of digital subtraction angiography (DSA) on the right internal carotid artery (ICA) injection.

**Figure 4:**
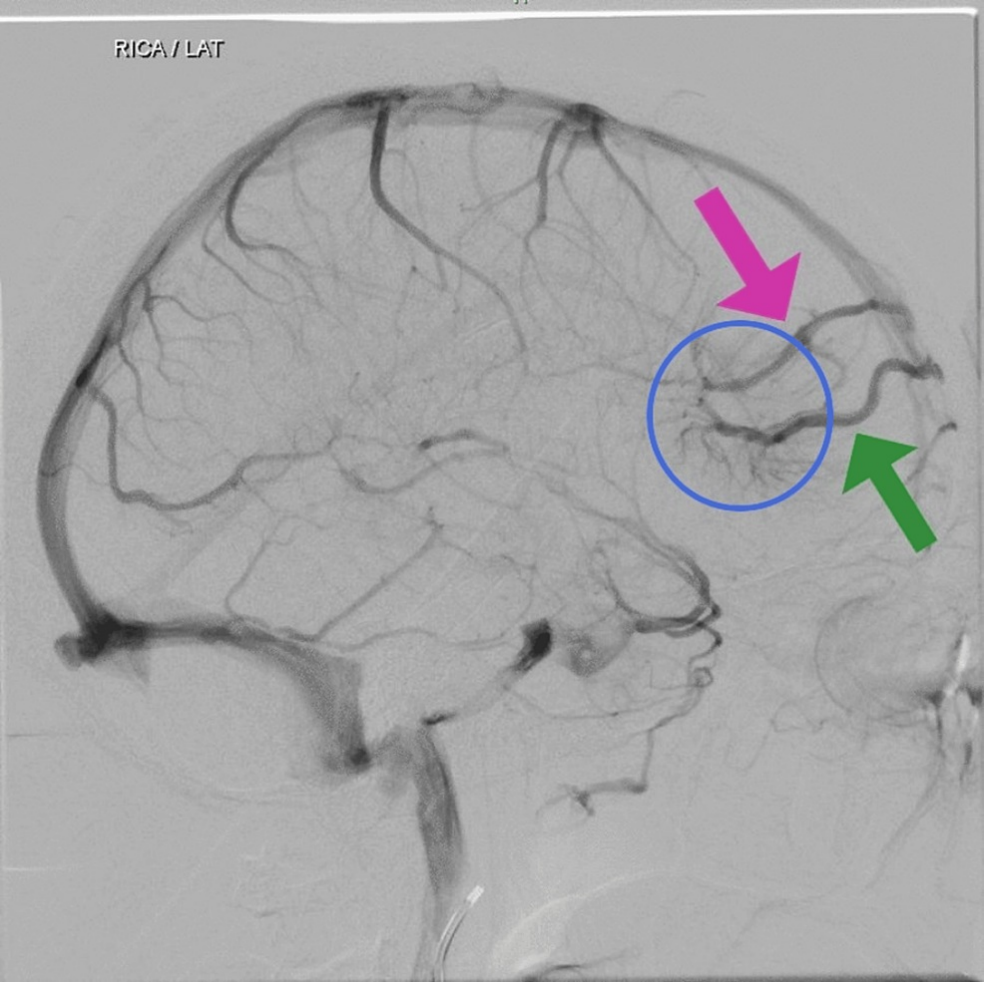
Caput medusa (blue circle) with two developmental venous anomalies (DVAs) are seen. The green arrow represents the common draining vein for the arteriovenous malformations (AVM) and one of the DVAs and the purple arrow represents the other DVA.

Cerebral digital subtraction angiography (DSA) was performed. It was accessed via the right common femoral artery and the catheter tip was placed in bilateral internal carotid artery (ICA), external carotid artery, and left vertebral artery. The right vertebral artery could not be cannulated due to tortuosity. A compact nidus AVM was noted in the right frontal region measuring 9 x 8 x 7 mm on anteroposterior (AP) and lateral view (Figure 2) with arterial feeder from the right M2 middle cerebral artery (MCA) branch and venous drainage via a cortical vein into superior sagittal sinus on AP and lateral view (Figure 3). Two DVAs were noted adjacent to the AVM, one of them sharing the same draining vein as the AVM (Figure 4). The Spetzler-Martin grading scale for intracranial arteriovenous malformations was one. In view of AVM with DVA, antiplatelet therapy was halted due to the risk of rupture, and the patient was advised endovascular treatment or gamma knife radiosurgery, however, the patient decided to continue treatment in his native country.

## Discussion

Central nervous system vascular malformations have classically been defined into four different groups namely the AVM, DVA, capillary telangiectasia, and cavernous malformation [8]. AVMs consist of clusters of arteries and veins that are deprived of an intervening capillary network, leading to arterio-venous shunting. Generally, normal brain tissues are present in between the abnormal blood vessels [9]. A DVA's angioarchitecture features a narrow vascular wall and limited compliance to changes in flow and pressure. Consequently, constriction of the vascular drainage can trigger localized venous hypertension, venous infarction, and bleeding. Conversely, an increase in DVA flow due to its connection with an arteriovenous shunt can also contribute to venous hypertension and bleeding. This subgroup of DVAs featuring arteriovenous shunts is identified by several terms, including predominantly venous parenchymal AVMs, venous angiomas with arterial supply, DVAs linked with capillary spots, and atypical DVAs. Pereira et al. adopted the term "arterialized DVAs" to describe this phenomenon [7]. DVAs with CVMs can co-exist together in one patient. These mixed vascular lesions have been reported to occur in as high as 33% of all CVMs however, DVAs with an AVM have the least reported incidence rate [10]. 

The etiology behind DVA is not well understood. Hussain et al. [11] and Mullan et al. [12] state that DVAs occur due to an arrest in the development of the venous system, resulting in the retention of primitive embryological medullary veins that drain into a single large vein. DVAs present as a spoke wheel appearance, wherein there are multiple deeper-located medullary veins that drain into superficial veins or a few deep dilated veins. On angiography, they are more accurately detected during the venous phase as one or more dilated draining veins, in comparison to the arterial phase, where they usually appear normal [11,13]. Despite previous speculations of a congenital etiology, a study by Brinjikji et al. has speculated an acquired etiology behind such vascular malformations. Their study showed that the prevalence of DVAs increased during the first 10 years of life suggesting its development as a result of adaptation of the cerebral venous system to thrombosis. They also reported a case of torcular AVM wherein multiple enlarging DVAs emerged with time as the AVMs progressed towards more aggressive angioarchitecture outcomes; highlighting that increased blood flow in the superficial venous system as a result of the AVM could be a potential cause behind the development of DVAs [14]. On the other hand, an opposing theory has been speculated behind its development. It has been proposed that AVMs develop as a consequence of DVAs due to thrombosis in the collector or medullary veins that can lead to the origination of fistulas [12].

Isolated DVAs without association with CVMs may in rare instances present with features of headache, dizziness, seizures, focal neurological deficits, and brain infarctions. These symptoms may result from increased inflow, restricted outflow, or compression of the neural tissue by the collector vein. A thorough assessment is essential when evaluating AVM via neuroimaging, and arteries with ectasia or those neighboring DVAs can be an indication of a shunt. For diagnosing arterialized DVAs, DSA stands as the definitive imaging method. While these lesions may resemble typical DVAs on MRI, catheter angiography can help visualize them better during their arterial phase [7].

A research article has classified DVAs into three unique categories based on their location deep, subcortical, and juxtacortical. When caput medusae are present, DVAs are categorized as superficial or deep. CT scans with contrast are needed for their detection where they appear as linear enhancing areas that radiate towards the ependymal surface of ventricles [15]. T1 and T2 weighted MRI exhibit features of absent flow signals at the site of DVAs with normal brain tissues adjacent to it. Multiple vessels emerge from the malformation in a curvilinear pattern giving the characteristic appearance of a medusa head on contrast-enhanced MRI. Similar findings are noted in the venous phase of cerebral angiography, wherein, multiple medullary veins converge into a dilated transcortical collector vein [9]. Im et al. angiographically described intracranial vascular malformations in 15 patients as atypical DVAs with arterio-venous shunts. This vascular malformation exhibited a fine arterial blush in the absence of a definitive nidus with an early filling of the dilated medullary vein that drained it during the arterial phase of angiography [16].

The current data on the benign character of isolated DVAs advocates for a more conservative management [17,18] as the likelihood of cerebral venous infarction occurring following the obliteration or excision of a DVA is widely known [19]. However, in cases where a DVA is present with an AVM, it is recommended to treat the AVM while preserving the DVA [9,18], as the DVA would play an important role in venous drainage from its localized area in the brain [19]. It is challenging to treat such malformations due to the resulting hemodynamic changes in the nearby normal tissue after the removal of the AVM [6]. The recommended treatments include surgery, radiosurgery, endovascular embolization, and combined therapy [9]. While partial obliteration may alter the hemodynamic conditions within the DVA, only complete nidus obliteration can prevent long-term re-bleeding [5]. 

According to a study, the global hemorrhage prevalence of AVM with DVAs is 38% and for isolated AVMs, it is 22.8% [5]. The estimated risk of hemorrhagic strokes in an isolated DVA is 0.15-0.68%. [7]. When DVAs occur in association with AVMs, they usually have more adverse outcomes such as hemorrhagic strokes when compared to isolated DVAs. Generally, complications such as hematoma, seizures, and headaches are to be treated [20]. The duration of follow-up in published case reports is inadequate to fully evaluate the long-term results [5].

## Conclusions

DVAs are symptomatic when they coexist with other CVMs. Thus, a thorough investigation becomes imperative to exclude any coexisting malformations, as management revolves around addressing the CVM while preserving the DVA. Patients with DVAs should undergo an MRI to exclude any other coexisting CVMs due to the increased risk of bleeding. We suggest physicians to consider CVM as a differential diagnosis when evaluating a patient with features suggestive of stroke and to rule out CVM before initiating antiplatelet therapy.
